# Impacts of social distancing during the covid19 pandemic on the development of children with autism in Brazil

**DOI:** 10.1192/j.eurpsy.2021.704

**Published:** 2021-08-13

**Authors:** P. Pacheco, M. Pacheco, D. Marinho, T. Oliveira, A. Marques, K. Souza, E. Franco, J. Maia, L. Silva, D. Molini-Avejonas

**Affiliations:** 1 Rehabilitation Science, USP, São Paulo, Brazil; 2 Morphology, UFES, Espirito Santo, Brazil

**Keywords:** autism, Development, social distancing, COVID19

## Abstract

**Introduction:**

COVID-19 is a respiratory disease and its main symptoms are fever, dry cough and difficulty breathing. It spread to several countries, which led the World Health Organization to decree, on March 11, 2020, a pandemic state that deeply affected Brazil. Due to the impossibility of leaving the house, the routine of children with autism was changed. Children in Autism Spectrum Disorder (ASD) have a qualitative deficit in social interaction. Clinical and daily observations reinforce several scientific studies that defend the importance of maintaining a routine as stable as possible for people with ASD, without this stability they may become emotionally disorganized, feel discomfort or even irritability.

**Objectives:**

Investigate the impact caused by social distancing on the development of children and adolescents with autism.

**Methods:**

An online questionnaire based on the DIR/Floortime basic map of emotional functional capacity development was distributed in Brazil from April to May, 2020. The results were analyzed using SPSS software.

**Results:**

Results obtained from 122 questionnaires showed that after 30 days of quarantine 20% of children no longer had the characteristic of being able to remain calm and organized for at least 2 minutes; 11% no longer initiates interactions with their parents; 27% demonstrated more protests and anger than before the social distancing; 18% demonstrated more emotions such as anger, fear and intimacy, 28% began to understand their limits and 12% of the children are using greater facial expression during the social distancing.

**Conclusions:**

This study brings results that can help to understand the processes in a child with autism.

